# Understanding the Relationship between Parental Psychological Control and Prosocial Behavior in Children in China: The Role of Self-Efficacy and Gender

**DOI:** 10.3390/ijerph191811821

**Published:** 2022-09-19

**Authors:** Wangqian Fu, Qianqian Pan, Weida Zhang, Lei Zhang

**Affiliations:** 1 Faculty of Education, Beijing Normal University, Beijing 100875, China; 2 Centre for Research in Pedagogy and Practice, Office of Education Research, National Institute of Education, Nanyang Technological University, Singapore 639798, Singapore; 3 School of Education, Renmin University of China, Beijing 100872, China; 4 Department of Politics, Beijing Health Vocational College, Beijing 100053, China

**Keywords:** parental psychological control, self-efficacy, prosocial behavior, gender, China

## Abstract

Objectives: Prosocial behavior is essential for individuals’ development, and the study aims to analyze the relationship between parental psychological control and prosocial behavior. Method: The current study investigated the relationships among Paternal Psychological Control (endogenous variable), General Self-Efficacy (mediator), and Prosocial Behaviors (exogenous endogenous variable) via a moderated mediation modeling approach (gender as the moderator). A total of 1822 Chinese students aged from 7- to 17-year-old (M_age_ = 12.4 years old, SD_age_ = 1.89, 48.6% girls) were included in the current study. Results: After controlling participants’ age, the only child status, family income, and parent’s education level, results revealed that higher levels of parental control were associated with lower levels of students’ self-efficacy, which, in turn, reduced students’ prosocial behavior intention. Moreover, the relationship between self-efficacy and prosocial behavior intention was moderated by students’ gender, where the positive effects of self-efficacy on prosocial behavior intentions were reduced in girls. Conclusion: Findings highlight the importance of parental psychological control for supporting children’s self-efficacy to promote prosocial behaviors on different gender groups.

## 1. Introduction

Prosocial behaviors are those with the intention to increase another person’s profit, which means a lot for human development. It can be defined as voluntary actions intended to help or benefit another individual or group of individuals [1]. Carlo and Randall (2002) define six types of prosocial behaviors, including public prosocial behavior, anonymous prosocial behavior, direct prosocial behavior, emotional prosocial behavior, compliant prosocial behavior, and altruistic prosocial behavior [2]. There is something in common that benefits other people.

Research on prosocial behavior has been conducted on different levels, including micro, meso, and macro levels. At the micro level, research focuses on searching for the root of prosocial behavior. At the meso level, research focuses on the individual, and the key question concerns the reasons and circumstances under which one helps. Meanwhile, the macro-level research examines the impact of prosocial behavior on groups, organizations, and society [3].

Studies at the meso level found that prosocial behavior is influenced by various factors, some of which are of a long-term character. One factor is previous experience, which is largely related to upbringing and social standards in the society in which a person grew up [4].

In the present study, we focused specifically on adolescents’ perceived parental psychological control. Parental psychological control refers to the attempt of parents to control the thoughts and feelings of children and adolescents by manipulating the emotional connection and intimate relationship between parents and children, including restricting verbal expression, uncertain feelings, withdrawal of love, and inducing guilt, etc. [5,6]. It seems intrusive and inhibits autonomy [7] and predicts increased internalizing problems for Finnish children [8].

However, some Western studies believe parental psychological control may function differently in other cultures [9,10,11], such as East-Asian cultures. Individuals in oriental society attach importance to the harmonious relationship with their surroundings and are willing to integrate into social relations. For that purpose, psychological control can be used to guide children’s behavior to be consistent with social values [9,10,12]. Thus, besides exploring the association of parental psychological control and children’ s prosocial behavior in Western culture, the mechanism between psychological control and children’s prosocial behavior is worthwhile exploring in East-Asian cultures.

### 1.1. Parental Psychological Control and Children’s Prosocial Behavior

Parents have historically been crucial to children and adolescents’ socialization [13]. Children’s perception of parental psychological control is critical in their growth, which may affect their adaptability [14]. It refers to parents’ control of their child’s emotional and psychological development through guilt induction, love withdrawal, and shaming [6], which is a known parenting strategy that limits children’s social-emotional development. Psychological control may be perceived in various ways. It is characterized by overprotection and intrusion [7] in Western countries and encouragement of independence and autonomy [15] in East-Asian cultures [9,10]. At the same time, a growing number of studies show that there is a positive association between psychological control and higher levels of socio-emotional problems, even in East-Asian cultures [16,17,18,19,20]. Besides, previous studies revealed that parental psychological control, including negative discipline exercises, such as humiliation, coercion, love withdrawal, and emotional manipulation, have been negatively related to prosocial behavior in previous studies [13]. A parent with a high level of psychological control might, for example, discredit how the child tries to solve problems and seize control when the child tries to initiate his or her own ideas. Psychological control is likely to alter the course of emotional and psychological adjustment. Thus, parenting represents an important proximal environmental factor that shapes long-term outcomes in children and may be an essential target for intervention.

### 1.2. Self-Efficacy and Prosocial Behavior

A lot of evidence shows that prosocial behavior is positively related to personal characteristics, including empathy, internalized moral reasoning, social anxiety, sympathy, collaboration, self-efficacy, and emotional regulation [21,22,23,24,25]. Self-efficacy is the perception of one’s capability to organize and execute required courses of action to achieve particular outcomes [26]. Individuals have basic motives to feel their own initiative, personal ability and ability, collective sense, or connection with others, and being valued by others [27,28,29]. Self-efficacy and social anxiety are the key variables reflecting individuals’ personal competence and connection to others.

From an agentic perspective, greater self-efficacy may increase prosocial behavior. Self-efficacy beliefs were found to predict some types of prosocial behavior (e.g., public), which may give adolescents the confidence to participate in prosocial behavior [30]. Moreover, academic self-efficacy has a positive association with prosocial behavior (Eklund et al., 2012) [31]. Caprara et al., (as cited in Bandura, 1993 [26]) found that children with a low sense of self-efficacy would have more physical and verbal aggression, while children with high self-efficacy would show more prosocial behavior and be less excluded by their peers [22]. When individuals feel efficacious in an activity, they are more willing to invest time and energy in it because they believe that their efforts can bring success [27,32].

Prior research suggests that higher parental psychological control is associated with a lower ability to adjust to new situations [33], which results in low self-efficacy to help others. Individuals often withhold help because they are uncertain about whether they will be able to help competently and effectively [34]. When helpers feel efficacious, they become more willing to provide help because they feel that their efforts will increase their odds of genuinely helping others [27].

Besides, even though issues of gender equality continue to be of great importance [35,36] and there has been progress for gender equality globally over time [37], social culture still has different role expectations for children of different genders [38]. In Asian culture, usually, boys are expected to be independent, brave, and tough. Thus, they prefer to control the situation. Meanwhile, girls are required to be friendly, considerate, demure, and gentle, and tend to pay attention to the interpersonal relationships [38,39]. Previous studies found males displayed a stronger sense of perceived ability, while females scored higher in prosocial behavior [40]. Therefore, it is worthwhile to examine the moderate effect of gender on the relationship between self-efficacy and prosocial behaviors.

### 1.3. The Current Study

Despite mounting evidence of adverse correlations between parental psychological control and negative correlations of prosocial behavior in Western culture, including U.S. [41], Canada [42], Israel [43], etc., relatively little work has been done to explicitly test whether associations between parental psychological control and prosocial behaviors are mediated by self-efficacy among children in China. Such information might help inform prevention and early intervention strategies to reduce parental psychological control and increase prosocial behavior among Chinese children. Because parents have historically been regarded as paramount to children and adolescents’ socialization, parenting is an essential factor. Further research is needed to characterize the parental psychological control influences on prosocial behavior and their mediating pathways [44]. To address the gaps, this study explores the association between parental psychological control and prosocial behavior and examines whether self-efficacy acts as a mediator in the Chinese context. Besides, the existing studies indicate that there may be gender differences in the relationship between self-efficacy and prosocial behavior. That is, the effect of self-efficacy on prosocial behavior may be moderated by gender. Therefore, this study also examined whether gender could impact the relationship between self-efficacy and prosociality. 

## 2. Methods

### 2.1. Participants and Procedure

We invited elementary school administrators who participated in a training program held in Beijing to help us deliver the questionnaire to students on a voluntary basis. The training was organized by the District School Board. The school administrators helped contact classroom teachers, and the classroom teachers helped get consent from students and their parents.

A total of 1822 students aged 7 to 17 (average age of 12.4 years old, SD of 1.89, 48.6% girls) participated in the current study. Table 1 presents the demographic information of participants. Among the participants, 56.1% were the only child in their households. 

### 2.2. Measures

Parental Psychological Control. Chinese Paternal Psychological Control Scale (CPPCS) and Chinese Maternal Psychological Control Scale (CMPCS) were used in the current study, which were developed by Shek (2007) for measuring parental psychological control in the Chinese social context [45]. CPPCS is composed of 10 items to measure father’s psychological control (e.g., “my father always wants to change my thoughts”). Identical items used in CMPCS to measure maternal psychological control (e.g., “my mother always wants to change my thoughts”). All items were scored on a 4-point Likert scale (1 = Strongly disagree, 2 = Disagree, 3 = Agree, 4 = Strongly agree). A higher score indicates a higher level of parental psychological control. 

Prosocial Behavior. The prosocial behavior scale comprises 5 items which were extracted by the Strengths and Difficulties Questionnaire (e.g., I am helpful if someone is hurt, upset or feeling ill), the items are rated on a 3-point rating scale (0 = not true, 1 = somewhat true, and 2 = certainly true). Higher scores represent a high degree of prosocial behavior [46]. A higher score indicates a higher level of prosocial behavior. Previous studies have demonstrated that the scale is an effective tool to measure prosocial behavior among adolescents, and it also has cross-cultural stability [47,48].

Self-efficacy. Self-efficacy was measured by using General Self-Efficacy Scale (GSES) [49]. It is a unidimensional scale, which is composed of 10 items for assessing perceived self-efficacy regarding coping and adaptation abilities (e.g., If I try my best, I will solve the problem eventually.). Item responses range from 1 (Strongly disagree) to 4 (Strongly agree). A higher score indicates a higher level of general self-efficacy. It has been shown good psychometric properties and is widely used internationally.

Control variables. Father and mother education levels (6-point scale from secondary school and below to doctoral degree), family income (9-point scale from ≤1000 RMB to >20,001 RMB), students’ gender, age, and single-child status were included in the model as control variables.

## 3. Data Analysis

### 3.1. Data Analysis Plan

Data analysis proceeded in three steps. Firstly, summary statistics of item responses were examined for all items. As shown in Table 2, item responses of CPPCS, CMPCS, and GSES had an acceptable range for being normally distributed, and item responses of Prosocial Behavior Measure did not satisfy the normality assumption [50].

Therefore, in the second step, the item responses of CPPCS, CMPCS, and GSES were treated as continuous. Thus, the latent construct of each scale was examined by applying the confirmatory factor analysis (CFA) using robust maximum likelihood estimation (MLR). Meanwhile, item responses to the Prosocial Behavior Measure were treated as categorical variables. Therefore, the diagonally weighted least squares (DWLS) estimator was used to examine the latent construct of these two questionnaires. All models were identified by setting the latent factor means to 0 and the variances to 1. Then, all item intercepts, item factor loadings, and item residual variances were freely estimated. The goodness of model fit was assessed as a Comparative Fit Index (CFI) value greater than 0.90 and a Root Mean Square Error of Approximation (RMSEA) value less than 0.08 [51]. McDonald’s omega was used to calculate the reliability of each CFA model [52]. 

Thirdly, after examining the latent structure of each scale, we computed the scale scores via the Empirical Bayes Modal approach provided in *Lavaan* package, and then we adopted a path analysis approach to test a series of hypothetic models to explore four research questions (RQs) as listed below:

RQ1: Does paternal and maternal psychological control influence students’ prosocial behavior intention?

RQ2: Does students’ self-efficacy mediate the relationship between parental and maternal psychological control and students’ prosocial behavior intention?

RQ3: Does students’ gender moderate the mediation effects of self-efficacy on parents’ control on prosocial behavior intentions?

Model 0: the baseline model, aimed to test the direct effects between parental control and students’ prosocial behavior intention after controlling parents’ education level, family income, students’ gender, age, and single-child status.

Model 1: the self-efficacy model, tested if self-efficacy mediated the relationship between students’ prosocial behavior intention and parental psychology control.

Model 2: the moderated mediation model, tested if gender moderated the mediation effects of self-efficacy on parental control and prosocial behaviors.

**Figure 1:**
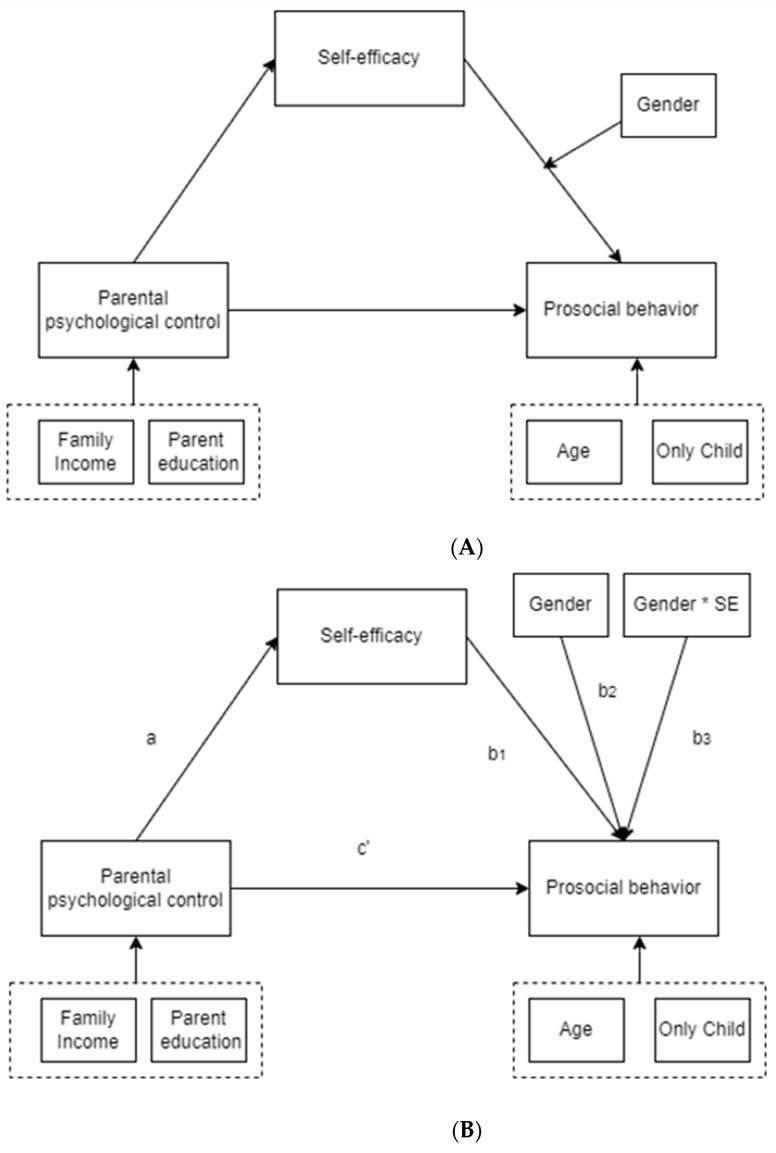
(**A**) diagrams of theoretical model; (**B**) diagrams of statistical model. Note. SE: self-efficacy; a: effect of parental psychological control on self-efficacy; b1: effect of self-efficacy on prosocial behavior; b2: effect of gender on prosocial behavior; b3: interaction effect of gender and self-efficacy on prosocial behavior; c′: effect of parental psychological control on prosocial behavior; * means a multiplication.

Theoretical diagrams are displayed in Figure 1 below.

### 3.2. Procedures for Testing Mediation Effects

Based on the literature review, we hypothesized that students’ self-efficacy (SE) could mediate the relationship between parental psychological control (ParCon) and students’ prosocial behavior intention (ProSoc). In addition, the effects of self-efficacy on prosocial behavior intention could be moderated by gender (See Figure 1A for a theoretical moderated mediation model diagram and Figure 1B for a statistical moderated mediation model diagram).

A mediation effect was estimated using the product of the coefficient method suggested by MacKinnon et al., (2002) [53]. As shown in Figure 1B, a confirmed mediation effect should meet the following criteria: (1) The endogenous factor had a statistically significant effect on the mediator (SE) (*a*), (2) the mediator (SE) had a statistically significant effect on the ProSoc (*b_1_*), (3) a statistically significant the mediated effects (*ab_1_*) [53]. In the mediation analysis, indirect effects are the production of the coefficients of the endogenous factor and the mediator. The direct effect is the coefficient between the endogenous factor and the exogenous factor. Therefore, the total effects are the sum of both indirect effects and direct effects. Bias-corrected confidence intervals with 1000 bootstrapped samples were used to test the indirect effects.

*ProSoc= c’ParCon + b*_1_*SE + z*_1_*ParEd + z*_2_*Income + z*_3_*Age + z*_4_*OnlyChild + e_ProSoc_* (1)

*SE = aParCon + e_SE_* (2)

where *ParCon*: Parental control, *SE*: Self-efficacy, *ProSoc*: Prosocial behavior intention, *ParEd*: Parent education level, *Income*: Family income, *OnlyChild*: Only child status, *e_ProSoc_* and *e_SE_*: Errors of each regression.

Furthermore, the moderated mediation model was specified in two regression equations, as shown below in Equations (3) and (4), to examine whether gender moderated the effects of self-efficacy on prosocial behavior intention. As pointed out by Preacher et al. (2007) [54], the indirect effects of ParCon on ProSoc through SE are a linear function of Gender (See Equation (5)), such that the weight for Gender (*ab_3_*) is the index of moderated mediation for this model, which is equal to the indirect effects. Bias-corrected confidence intervals with 1000 bootstrapped samples were used to test the indirect effects.

*ProSoc= c’ParCon + b*_1_*SE + b*_2_*Gender + b*_3_*SE × Gender + z*_1_*ParEd + z*_2_*Income + z*_3_*Age + z*_4_*OnlyChild + e_ProSoc_* (3)

*SE = aParCon + e_SE_* (4)

*w = a(b*_1_*+ b*_3_*Gender) = ab*_1_* + ab*_3_*Gender* (5)

The analyses were conducted in the programming environment R using *lavaan*.

## 4. Results

### 4.1. Latent Factor Structure and Reliabilities

Paternal psychological control scale. Based on the confirmatory factor analysis (CFA), a single-factor construct was supported by acceptable model fit statistics and reliability (ω*_PPCS_* = 0.90). Maternal psychological control scale. The same single-factor construct of MPCS as PPCS was fit to the data and achieved acceptable model fit statistics and reliability (ω*_MPCS_* = 0.93). Self-efficacy scale. Based on the literature, a single-factor model was fit to the data. The model achieved acceptable model and reliability (ω*_SE_* = 0.82). The summary of model fit and reliabilities was shown in Table 3.

### 4.2. Mediation Effects Testing

A total of six models were conducted to answer the research questions listed above. As shown in Table 3, all CFA models achieved good model fit by the CFI and TLI values being larger than 0.95 and RMSEA values being lower than 0.05, which allowed us to export the factor scores and then test mediation models.

Table 4 presents the correlations among targeted variables used in the mediation study. As expected, students’ self-efficacy was significantly correlated to parental control and prosocial behavior intentions (*r_SE__−FaCon_* = −0.14, *p* < 0.01; *r_SE−MoCon_* = −0.21, *p* < 0.01; *r_SE−ProSoc_* = 0.43, *p* < 0.01; *r_ProSoc__−FaCon_* = −0.10, *p* < 0.01; *r_ProSoc__−MoCo_* = −0.11, *p* < 0.01). As evidenced by previous studies and these medium-to-large correlation relationships, it is reasonable to infer that students’ self-efficacy might mediate the relationship between their parental control and their prosocial behavior intentions. 

Furthermore, gender was also correlated with prosocial behavior intentions to a lesser but significant extent (*r_female__−ProSoc_* = 0.06, *p* < 0.05), which suggested further investing any gender effect on prosocial behavior intentions and mediation effects of students’ self-efficacy. Together, the correlations suggested further investigating the mediation effects.

However, as shown in Table 4, Paternal psychological control and maternal psychological control were highly correlated (*r_FaCon__−MoCon_* = 0.068, *p* < 0.01), indicating collinearity. When highly correlated variables are included in the mediation analysis simultaneously, it might result in nonsignificant direct effects or unexpected sign changes (Hair et al., 2017) [55]. Therefore, we examined these Paternal psychological control and Maternal psychological control separately, where Paternal Model only included paternal psychological control and the maternal model only included maternal psychological control. 

Table 5 presents the standardized coefficients of mediation analyses. Paternal Model 0 reveals that fathers’ education level and family income did not influence their psychological control. Students’ prosocial behavior intention is significantly associated with their ages. More specifically, students’ prosocial behaviors decreased as aging. Furthermore, paternal control has a significant negative influence on students’ prosocial behavior intention above and beyond students’ demographic variables. Paternal Model 1 shows that paternal control is significantly negatively associated with students’ self-efficacy (*a_fa_* = −0.15, *p* < 0.001) and students’ self-efficacy is significantly positively related to students’ prosocial behavior intention (*b_1fa_* = 0.36, *p* < 0.001). Additionally, a significant indirect effect of paternal control on students’ prosocial behavior intention through students’ self-efficacy (*a_fa_b_1fa_* = −0.05, *p* < 0.001) is detected. Moreover, the direct effects of paternal control on students’ prosocial behavior intention were no longer significant, which indicates students’ self-efficacy fully mediated this relationship.

Moreover, Paternal Model 2 revealed that female students had higher levels of prosocial behavior intentions in general ($b_{2fa}=0.10,\;p<0.001$). In addition, student’s gender moderated the impacts of individual self-efficacy on prosocial behavior intentions, which was supported by the significant interaction effects between gender and self-efficacy ($b_{3fa}=-0.11,\;p<0.001$). Therefore, the effect of self-efficacy on prosocial behavior was 0.42 for males and 0.32 for females, indicating that male students’ prosocial behavior intentions were more influenced by their self-efficacy. Then, the total effects of paternal control on female students’ prosocial behavior intentions were −0.08, and the total effects of paternal control on male students’ prosocial behavior intentions were −0.10.

In terms of the impacts of maternal psychological control, similarly, Maternal Model 0 shows that the mother’s education level and family income were not significantly associated with maternal control levels. Furthermore, Maternal Model 1 also reveals that maternal control is significantly negatively associated with students’ self-efficacy ($a_{mo}=-0.22,\;p<0.001$), students’ self-efficacy is positively associated with their prosocial behavior intention ($b_{mo}=0.36,\;p<0.001$), such that students’ self-efficacy mediated the relationship between maternal control and students’ prosocial behavior intention ($a_{mo}b_{1mo}=-0.08,\;p<0.001$). Similarly, students’ self-efficacy fully mediated the relationship between maternal control and their prosocial behavior intention. Maternal model 2 has the same moderation effects of gender on the impacts of self-efficacy on prosocial behavior. However, the total effects of maternal control on female students’ prosocial behavior intentions were −0.09, and the total effects of maternal control on male students’ prosocial behavior intentions were −0.11, which was slightly larger than the paternal model. See Table 6 for the summary of mediation effects with the confidence intervals.

Together, the moderated mediation analysis from both paternal and maternal model 2 showed that female students had higher levels of prosocial behaviors in general than findings from previous studies suggest. However, the current analyses show that male students who had higher levels of self-efficacy could make up this gender gap, which was supported by a larger positive effect of self-efficacy on prosocial intentions.

## 5. Discussion

The study aimed to understand the relationship between parental psychological control and prosocial behavior in children and adolescents. Understanding the influences of parenting styles on child development is a critical task of developmental psychology [56], and prosocial behavior is a vital component of developmental goals with a composite and multidimensional construct [57]. Due to this importance and omnipresence, prosocial behavior has received considerable attention around the world [58].

First, we found that students’ prosocial behavior intention is significantly negatively associated with parental psychological control. According to developmental psychologists [59], parenting styles are essential in culturing prosocial behaviors in adolescents. The social and cognitive models of prosocial behaviors suggest that parenting style and socioemotive and sociocognitive development have a great impact on teenagers’ prosocial behaviors [60]. Parental psychological control is an important aspect of parenting style, and it has been recognized by a majority of developmental psychologists [17]. Psychologically controlling parents use intrusive and manipulative strategies, such as isolation, inducing guilt, constraining, invalidating, shaming, and withdrawal of affection to make children meet their expectations and change children’s opinions, emotions, and behavior patterns [61]. Existing prior studies from Western cultures have demonstrated the associations between parental psychological control and internalizing symptoms and problem behavior among adolescents, such as cyberbullying victimization [62], anxiety [63], depression, suicidal ideation [64], and social withdrawal [65]. Meanwhile, these internalizing symptoms and problem behavior will decrease prosocial behavior. In addition, results showed that students’ prosocial behavior intentions were not significantly associated with their ages and only child status. Although a large number of studies reported changes in prosocial behavior across the age range in adolescence [66], our studies did not support this previous finding, which might indicate that studies on the relationship between age and prosocial behaviors are not comprehensive nor inconclusive yet.

Second, family income and parents’ education level did not influence their psychological control. Although previous research found that mothers with higher levels of education are positively associated with positive parenting and negatively associated with harsh parenting [67], our studies did not support this finding. This finding might indicate that parental psychological control might be different from other negative parenting behaviors as well as the complexity of the relationship between parenting backgrounds and parenting behaviors. Therefore, parents with or without higher levels of education might not be necessarily associated with one type of parenting.

Thirdly, the results from the mediation model showed that the relationship between parental psychological control and students’ prosocial behavior intentions was fully mediated by students’ self-efficacy, where a higher level of parental psychological control was associated with a lower level of student’s self-efficacy, and then, in turn, a lower level of prosocial behavior intentions. Our results support the person–context interaction theories [68,69], which regard people and situations as an interactive interaction as a whole. The situation will affect people’s psychological system and then influence individual behavior, namely situation → intermediary psychological system (know perception, cognition, emotion/affective system) → individual behavior. In this study, parental psychological control is an important situational factor, the sense of efficacy corresponds exactly to the factors mediating the psychological system, and prosocial behavior is the individual behavior. Parental control has been found to have an important effect on self-efficacy in many studies [70,71]. A good family environment gives children more attention and support, which results in higher self-efficacy [57,72]. Meanwhile, various studies suggest that self-efficacy is determinant of prosocial behavior [22,73]. In line with our expectations, the results provide novel information that parental psychological control was negatively associated with self-efficacy, supporting evidence that parental psychological control is related to lower adolescent refusal self-efficacy [74]. Parents who use psychological control treat their children as extensions of themselves rather than as separate individuals, making it difficult to develop children’s self-efficacy [20,75]. That is, parental psychological control decreases self-efficacy, leading to negative effects on teenagers’ prosocial behavior.

In addition, we found that gender moderated the association between self-efficacy and children’s prosocial behavior. The study reveals that girls report higher levels of prosocial behavior than boys [23,66]. Furthermore, the gender differences in the effect of self-efficacy on prosocial behavior intentions were different across genders. Boys had a larger positive influence of self-efficacy on prosocial behavior intentions. It may be due to the gender-differentiated nature of children’s peer relations, whereby boys pay more attention to the control in the relationship [22], while girls’ relationships are more interpersonal by nature [76]. Results in the present study also echo the claim that self-efficacy plays a more important role in boys´ behavior from a previous study [77].

Overall, the study examined the mediation effect of self-efficacy between parental psychological control and prosocial behavior, which distills the mechanisms through which parental psychological control hampers prosocial behavior and analyzed the role of gender in the relationship between self-efficacy and prosocial behavior. In conclusion, our research contributes to a growing body of evidence supporting that parental psychological control will predict decreased prosocial behavior. Additionally, contemporary developmental theory stresses the interaction between different parenting variables and the interaction between parenting and SES.

## 6. Limitations and Further Research

Limitations of our findings include their subjective and cross-sectional nature. First, our study only relies on a self-reported approach to measuring prosocial behaviors in a single situation. As a result, this might have been partially biased, for participants may embellish their actions. Future studies could collect information from more objective sources. Second, the cross-sectional research also limits the possibility of interpreting the directionality of the relations. Longitudinal investigations on this topic will be useful in further study. In addition, although we found significant indirect effects in two moderated mediation models, the effect sizes appeared small, which raised concerns about the practical meaningfulness of our findings. Furthermore, there has been an ongoing discussion about whether the detrimental effects of psychological control techniques are culture-dependent. Studies with a larger sample across different cultures are necessary to confirm the results of our current study.

## 7. Conclusions

The present study found that higher levels of parental control were associated with lower levels of students’ self-efficacy, which, in turn, reduced students’ prosocial behavior intention after controlling participants’ age, the only child status, family income, and parent’s education level. Moreover, students’ gender moderated the relationship between self-efficacy and prosocial behavior intention was moderated, where the positive effects of self-efficacy on prosocial behavior intentions were reduced in girls. In sum, these findings highlight the importance of parental psychological control for supporting children’s self-efficacy to promote prosocial behaviors on different gender groups.

## Figures and Tables

**Table 1:** Summary of Demographic Information.

Variables	N	%
Gender		
male	937	51.40
female	885	48.60
Only child		
Yes	1022	56.09
No	800	43.90779
Rank		
1	475	59.38
2	278	34.75
3	37	4.63
4	10	1.25
Father education		
secondary school and below	427	23.44
high school	743	40.78
junior college	319	17.51
bachelor	237	13.01
master	12	0.66
doctoral	2	0.11
missing	82	4.50
Mother education		
secondary school and below	435	23.87
high school	676	37.10
junior college	354	19.43
bachelor	260	14.27
master	10	0.55
doctoral	5	0.27
missing	82	4.50
Family income (RMB)		
≤1000	28	1.54
1001–1500	39	2.14
1501–2000	32	1.76
2001–3000	100	5.49
3001–5000	310	17.01
5001–8000	431	23.66
8001–10,000	351	19.26
10,001–20,000	337	18.50
>20,001	112	6.15
missing	82	4.50

**Table 2:** Summary Statistics of Item Responses.

Scale	Item Number	Mean	SD	Skew	Kurtosis
Paternal psychological control	1	2.16	0.79	0.21	−0.48
	2	2.14	0.81	0.32	−0.39
	3	2.14	0.83	0.30	−0.51
	4	1.94	0.77	0.61	0.14
	5	2.21	0.90	0.26	−0.74
	6	2.05	0.82	0.43	−0.34
	7	1.94	0.78	0.57	0.02
	8	1.92	0.77	0.57	0.02
	9	1.76	0.71	0.78	0.71
	10	2.01	0.81	0.48	−0.25
Maternal psychology control	1	2.08	0.81	0.33	−0.46
	2	2.08	0.81	0.31	−0.52
	3	2.08	0.82	0.32	−0.55
	4	1.91	0.75	0.60	0.19
	5	2.14	0.86	0.33	−0.58
	6	1.96	0.80	0.54	−0.15
	7	1.95	0.80	0.52	−0.24
	8	1.93	0.78	0.54	−0.12
	9	1.86	0.76	0.65	0.12
	10	2.05	0.83	0.38	−0.52
Prosocial behavior	1	2.45	0.60	−0.60	−0.58
	2	2.50	0.60	−0.73	−0.45
	3	2.58	0.59	−1.05	0.10
	4	2.57	0.60	−1.07	0.12
	5	2.51	0.59	−0.76	−0.40
Self-efficacy	1	2.84	0.85	−0.17	−0.76
	2	2.41	0.87	0.34	−0.58
	3	2.17	0.91	0.43	−0.59
	4	2.42	0.88	0.26	−0.65
	5	2.23	0.85	0.46	−0.32
	6	2.80	0.89	−0.10	−0.93
	7	2.62	0.89	0.10	−0.85
	8	2.55	0.85	0.22	−0.68
	9	2.70	0.84	0.12	−0.84
	10	2.41	0.86	0.27	−0.56

**Table 3:** Summary of Model Fit and Reliabilities.

	CFI	TLI	RMSEA	90% RMSEA Interval		Omega
				Lower	Upper	
Paternal psychological control	0.96	0.95	0.08	0.07	0.09	0.90
Maternal psychological control	0.97	0.95	0.09	0.08	0.09	0.93
Prosocial behavior	1.00	1.00	0.01	0.00	0.04	0.95
Self-efficacy	0.96	0.95	0.07	0.07	0.08	0.87

**Table 4:** Correlations of Targeted Variables.

	SE	FaCon	MoCon	ProSoc	Female	Age	OnlyChild	FaEdu	MoEdu
SE									
FaCon	−0.14 **								
MoCon	−0.21 **	0.68 **							
ProSoc	0.43 **	−0.10 **	−0.11 **						
Female	−0.03	−0.07 **	0.01	0.06 *					
Age	−0.06 **	−0.02	−0.03	−0.07 **	−0.04 **				
OnlyChild	−0.01	0.03	−0.01	0.03	−0.12 **	0.02			
FaEdu	0.06 *	−0.03	−0.04	0.04	−0.01	−0.03	0.21 **		
MoEdu	0.07 *	−0.03	−0.05	0.04	−0.01	−0.07 **	0.26 **	0.68 **	
Income	0.06 *	−0.01	−0.01	0.05 *	−0.04	<0.01	0.17 **	0.38 **	0.36 **

Note. * *p* < 0.05, ** *p* < 0.01. SE: Self-efficacy; FaCon: Paternal psychological control; MoCon: Maternal psychological control; ProSoc: Prosocial behavior; FaEdu: Father education level; MoEdu: Mother education level; Age: students’ age; OnlyChild: Only Child in the household.

**Table 5:** Summary of Mediation Effects.

		Model 0			*R^2^*	Model 1			*R^2^*	Model 2			*R^2^*
		Std.Coef(SE)	95% CI			Std.Coef(SE)	95% CI			Std.Coef(SE)	95% CI		
Paternal Model													
ProSoc	FaCon	−0.09(0.02) **	−0.13	−0.04	10.4%	−0.04(0.02)	−0.08	0.02	19%	−0.03(0.02)	−0.09	<0.01	24%
	OnlyChild	0.04(0.04)	−0.06	0.11		0.04(0.04)	−0.05	0.10		0.04(0.04)	−0.03	0.12	
	Age	−0.02(0.01) *	−0.03	<0.01		−0.01(0.01)	−0.03	0.01		−0.01(0.01)	−0.02	0.01	
	SE					0.36(0.02) **	0.32	0.41		0.42(0.03) **	0.36	0.47	
	Female									0.1(0.04) **	0.04	0.17	
	SE × Female									−0.11(0.04) **	−0.19	−0.06	
FaCon	Income	<0.01(0.02)	−0.03	0.04	<0.01%	0(0.02)	−0.04	0.04	<0.01%	0(0.02)	−0.03	0.03	<0.01%
	FaEdu	−0.03(0.03)	−0.09	0.03		−0.03(0.03)	−0.07	0.07		−0.03(0.03)	−0.11	0.02	
SE	FaCon					−0.15(0.03) **	−0.23	−0.10	2%	−0.15(0.03) **	−0.22	−0.09	2%
Maternal Model													
ProSoc	MoCon	−0.1(0.02) **	−0.14	−0.06	2%	−0.02(0.02) **	−0.06	0.02	18%	−0.02(0.02)	−0.07	0.01	24%
	OnlyChild	0.03(0.04)	−0.04	0.11		0.03(0.03)	−0.02	0.12		0.04(0.04)	−0.03	0.12	
	Age	−0.02(0.01) *	−0.03	0.00		−0.01(0.01)	−0.02	0.01		−0.01(0.01)	−0.02	<0.01	
	SE					0.36(0.02)	0.32	0.39		0.42(0.03) **	0.37	0.48	
	Female									0.11(0.03) **	0.06	0.18	
	SE × Female									−0.11(0.04) **	−0.19	−0.03	
MoCon	Income	0.01(0.02)	−0.03	0.04	<0.01%	0.01(0.02)	−0.02	0.04	<0.01%	0.01(0.02)	−0.02	0.04	<0.01%
	MoEdu	−0.05(0.03)	−0.09	0.03		−0.05(0.03)	−0.10	0.02		−0.05(0.03)	−0.10	<0.01	
SE	MoCon					−0.22(0.03) **	−0.27	−0.16	5%	−0.22(0.03) **	−0.27	−0.16	5%

Note. FaCon: Paternal control; MoCon: Maternal control; ProSoc: Prosocial behavior intention; SE: Self-efficacy; MoEdu: Mother education level; FaEdu: Father education level; SE × Female: Interaction between students’ self-efficacy and gender *: p < 0.05; **: *p* < 0.01.

**Table 6:** Summary of Mediation Effects of Self-efficacy between Parental Control and Students’ Prosocial Behavior Intention.

	Paternal Model 2		Maternal Model 2	
	Indirect Effects (95% CI)	Total Effects (95% CI)	Indirect Effects (95% CI)	Total Effects (95% CI)
	−0.05 (−0.09, −0.04)	−0.09 (−0.14, −0.05)	−0.08 (−0.10, −0.06)	−0.10 (−0.14, −0.05)
Female	−0.05 (−0.08, −0.03)	−0.08 (−0.13, −0.04)	−0.07 (−0.09,−0.04)	−0.09 (−0.13, −0.04)
Male	−0.06 (−0.10, −0.04)	−0.10 (−0.16, −0.05)	−0.09 (−0.12,−0.07)	−0.11 (−0.15, −0.07)

## Data Availability

The datasets generated during and analyzed during the current study are available from the corresponding author on reasonable request.
